# Letters to a Candid Inquirer on Animal Magnetism

**Published:** 1851-11

**Authors:** 


### ARTICLE V.

*Letters to a candid inquirer, on Animal Magnetism.* By William Gregory, M. D., F. R. S. E., Professor of Chemistry in the University of Edinburgh. pp. 384, duodecimo. Philadelphia, Blanchard & Lee, 1851. (From the Publishers, through S. C. Griggs & Co.)

From a hasty glance at this work we infer that it is a defence of the so called Science of Animal Magnetism; but as the book has but just come to hand we are unable for want of time at present to give its contents an analysis.

There is so much claimed for Animal Magnetism, by its advocates, that makes too strong a demand on our credulity, that many of us are inclined to reject the whole affair altogether, And yet there are some manifestations that would seem to show that there is an influence exerted by its manifestations upon certain subjects, that is worthy of serious investigation. The most satisfactory explanation of these, that we have seen, refers the physical phenomena manifested to the influence of the mind ; which seems to be corroborated by the fact, that the susceptible subjects are imaginative, speculative and nervous people.

To those who are curious on the subject, the work before us offers the attractions of a distinguished author, and a systematic arrangement of the topics discussed.

